# Bridging the translational gap in systems neuroscience: from circuit mechanisms to clinical therapeutics

**DOI:** 10.3389/fphar.2026.1791688

**Published:** 2026-03-06

**Authors:** Anyin Wang, Jiale Ye, Jie Li, Xiaoqin Chen, Qiaoyan Wang, Kunwei Wu

**Affiliations:** 1 Department of Gastrointestinal and Hernia Surgery, Chongqing Liangping District People’s Hospital, Chongqing, China; 2 Jiangsu Province Key Laboratory of Anesthesiology and Brain Science, NMPA Key Laboratory for Research and Evaluation of Narcotic and Psychotropic Drugs, School of Anesthesiology, Xuzhou Medical University, Xuzhou, Jiangsu, China; 3 Administrative Office, Chongqing Liangping District People’s Hospital, Chongqing, China; 4 Department of Anesthesiology, The Affiliated Hospital of Xuzhou Medical University, Xuzhou, Jiangsu, China

**Keywords:** deep brain stimulation, neural network, neuromodulation, optogenetics, therapeutics, translational neuroscience

## Abstract

The advent of optogenetics, chemogenetics, and high-density neural recording technologies has propelled systems neuroscience into a golden age, generating unprecedented mechanistic insights into how defined neural circuits orchestrate behaviour. These tools have allowed us to move beyond correlational observations to establish causal links between specific circuit dynamics and behavioural states. However, a profound and disheartening translational dilemma has emerged: the pace at which these foundational discoveries in model organisms have yielded novel, effective therapeutics for human neuropsychiatric disorders remains glacial. This review argues that this dilemma is not a failure of the science itself but a consequence of a multi-layered gulf between basic discovery and clinical application. This gulf encompasses technological, phenomenological, and biological disparities. We analyse the roots of this impasse and propose a concerted, multi-pronged strategy to bridge it, focusing on back-translation, cross-species behavioural dimensionalization, the development of non-invasive neuromodulation, and the fostering of deeply integrated interdisciplinary collaborations. The path forward requires a fundamental shift in how we design, interpret, and prioritize neural circuit research with translation in mind.

## 1 Introduction

The past two decades have witnessed a revolutionary transformation in neuroscience, driven by the development of powerful tools that enable precise observation and manipulation of neural circuits in behaving animals. The emergence of optogenetics has been particularly transformative, allowing researchers to establish causal relationships between activity in genetically defined neuronal populations and behavior by using light to control neural activity with millisecond precision (Deisseroth, 2015). Concurrent advances in neural recording technologies, including fibre photometry and high-density silicon probes, have enabled researchers to observe ensemble circuit dynamics with unprecedented temporal and spatial resolution (Russell et al., 2022; Rajasethupathy et al., 2016). These methodological breakthroughs have generated remarkably detailed mechanistic models of brain function and dysfunction, successfully delineating the roles of specific neural pathways in various processes (Yuste et al., 2024; Akiki et al., 2025; Mota and Madden, 2024; Li H. et al., 2025).

This accumulation of detailed circuit-level knowledge has generated legitimate excitement and anticipation within the field. For the first time, researchers have established a causal, circuit-level framework for understanding the neural basis of psychiatric disorders including depression, anxiety, and addiction. The logical expectation following these discoveries was that identifying dysfunctional circuits would directly reveal precise therapeutic targets for intervention (Fenster et al., 2018; Melle et al., 2025). However, this promise of therapeutic translation has remained largely unrealized. Despite exponential growth in our mechanistic understanding, clinical progress has been disappointingly slow. The development of novel pharmacological treatments for psychiatry has stagnated, with few truly innovative mechanisms reaching patients in recent decades. While neuromodulation techniques, particularly Deep Brain Stimulation (DBS), have demonstrated clinical efficacy for certain conditions, their application remains largely anatomically broad and empirically guided, lacking the cell-type specificity and circuit-level precision routinely demonstrated in preclinical studies (Krauss et al., 2021; Roet et al., 2020).

This profound disconnect between sophisticated mechanistic understanding and limited clinical progress can be quantified as a striking publication-to-therapy chasm. As detailed in Table 1, technologies like optogenetics and chemogenetics have generated thousands of high-impact publications that have fundamentally revolutionized our understanding of brain circuits, yet their direct clinical application remains negligible. Functional neuroimaging, while ubiquitous in human neuroscience research, has yielded predominantly descriptive correlations with limited success in identifying actionable therapeutic targets (Poldrack et al., 2017). Notably, the most clinically successful neuromodulation approach—DBS—was developed through empirical clinical observation rather than hypothesis-driven circuit neuroscience, highlighting the historical divergence between therapeutic development and mechanistic discovery (Benabid et al., 2009; Lozano and Lipsman, 2013). The translation gap therefore stems not from insufficient scientific activity, but from fundamental challenges in bridging the “valley of death” between mechanistic discovery in controlled experimental settings and the development of safe, effective, and regulatable interventions for heterogeneous human populations.

**TABLE 1:** Translational landscape of optogenetics and chemogenetics.

Technique	Foundational research output	Direct clinical breakthroughs	Translational status and barrier
Optogenetics	>14,000 PubMed records since ∼2005	∼2–5 early-phase therapeutic avenues (e.g., retinal gene therapy) (Sahel et al., 2021; Brar et al., 2024)	Status: Struggling in early trials. Barrier: Invasive gene-and-device therapy; major safety/regulatory hurdles
Chemogenetics (DREADDs)	>3,900 PubMed records since ∼2005	0 approved therapies; 0 active clinical trials	Status: Preclinical tool. Barrier: Requires viral gene delivery and novel ligand pharmacology; no clear path to human use

The central translational dilemma of modern neuroscience thus involves navigating a multidimensional challenge: bridging fundamental biological differences in brain complexity across species (Hodge et al., 2019), overcoming the limited construct validity of current diagnostic categories, and developing technical frameworks capable of translating precise circuit manipulations into clinically viable interventions. This review aims to analyze the roots of this translational impasse and propose an integrated strategic framework for progress. By critically examining limitations across experimental, conceptual, and clinical domains, and by advocating for a more synergistic approach that aligns discovery science with translational engineering, we seek to provide a roadmap for accelerating the transformation of circuit-level insights into meaningful therapeutic advances for neuropsychiatric disorders.

## 2 The anatomy of a dilemma: a multi-layered gulf

The translational impasse in systems neuroscience is not a single barrier but a multi-layered gulf formed by interconnected gaps in technology, methodology, and biological understanding. While tools like optogenetics have delivered transformative causal insights into brain circuits in animal models, the path from these discoveries to effective, scalable human therapies remains obstructed. To navigate this chasm, we must first precisely map its contours.

### 2.1 The spatiotemporal resolution gulf

A primary, quantifiable barrier is the profound mismatch in spatiotemporal resolution between our most powerful discovery tools and our clinical intervention platforms. This fundamental discrepancy is summarized in Table 2, which compares key parameters—such as spatial precision, temporal resolution, and causal specificity—across major experimental and clinical techniques. The contrast between the cellular/millisecond precision of research tools (e.g., optogenetics) and the regional/crude temporal dynamics of clinical tools (e.g., TMS, DBS) creates a fundamental interpretive problem, often rendering promising circuit-based targets “un-translatable” in practice. In the laboratory, techniques like optogenetics and chemogenetics offer cellular specificity (targeting genetically defined neuronal populations) and millisecond-scale temporal precision, allowing researchers to mimic natural neural activity patterns. In contrast, established clinical neuromodulation tools such as Transcranial Magnetic Stimulation (TMS) or even invasive Deep Brain Stimulation (DBS) operate with regional specificity (affecting mixed cell types within a brain area) and crude temporal dynamics (e.g., continuous or slow rhythmic stimulation), which can introduce non-physiological network states (Lozano et al., 2019; Hallett, 2007).

**TABLE 2:** Translational gulf in key neuroscientific techniques.

Technique	Primary use	Spatial precision	Temporal precision	Key translational barrier
Optogenetics	Animal: causal manipulation	Cell-type specific (µm)	Millisecond	Invasive; requires genetics and implants
fMRI	Human: systems Imaging	Regional (∼mm)	Slow (∼1–3 s)	Indirect signal; poor temporal resolution
TMS	Human: non-Invasive Modulation	Gross Cortical (cm)	Crude (s to min)	Low spatial precision; superficial targets only
DBS	Human: Invasive Therapy	Focal (mm)	Continuous/Patterned	Invasive surgery; affects mixed cell types

This discrepancy has direct and consequential clinical implications. For instance, while acute, cell-type-specific inhibition of the ventral tegmental area (VTA) dopamine neurons can robustly suppress reward-seeking behavior in rodent models of addiction, clinical attempts to modulate this circuit in patients—using broader, less specific DBS—have yielded inconsistent efficacy and significant side effects (Kuhn et al., 2014). The preclinical intervention targets a precise causal node, but the clinical tool engages a heterogeneous neural population, potentially activating compensatory pathways or disrupting normative functions. Similarly, the success of optogenetically silencing a specific basolateral amygdala projection to reduce conditioned fear in mice has not directly translated into reliable, targeted therapies for human anxiety disorders, highlighting the challenge of applying a precise “on/off” logic to a complex, distributed human emotional network (Tye et al., 2011; Langevin et al., 2016).

A second, more fundamental translational challenge lies in the observational gap between animal and human measurement techniques, creating a profound difficulty in cross-species mapping of circuit mechanisms. Basic animal research, typically utilizing single-unit recordings or calcium imaging, captures neuronal activity with millisecond-scale temporal precision and micrometer spatial resolution (Chen et al., 2013; Jun et al., 2017). Conversely, standard non-invasive human neuroimaging techniques operate at vastly different scales: functional magnetic resonance imaging (fMRI) measures the Blood Oxygenation Level Dependent (BOLD) signal—an indirect metabolic correlate—integrating signals over seconds and pooling activity across millions of neurons, with a spatial resolution typically in the millimeter range (Logothetis, 2008). While (electroencephalography and magnetoencephalography) EEG/MEG provides superior temporal resolution (milliseconds), it suffers from poor spatial resolution, particularly for deep brain structures (Baillet, 2017). This fundamental mismatch in spatiotemporal scales is further exacerbated by the inherent difference in the measured signals—direct electrical activity (spikes/LFPs) in animal models versus metabolic correlates (BOLD) in humans. This gulf makes it exceedingly difficult to confirm that the circuit dysfunction observed at the precise neuronal level in animal models is accurately captured by an accessible clinical imaging biomarker.

This technological disparity is compounded by the non-physiological nature of many current circuit manipulation approaches. The high-intensity, synchronous activation or inhibition of neuronal populations often employed by first-generation optogenetics or chemogenetics diverges significantly from the brain’s natural sparse, asynchronous firing patterns and subtle modulations (Huber et al., 2008; Otchy et al., 2015). Such “on/off” interventions risk generating artificial behavioral outputs or states that may not genuinely reflect the target circuit’s pathological function (Huber et al., 2008; Otchy et al., 2015). Crucially, these simplified, acute manipulations often fail to account for the brain’s inherent robustness and redundancy (Turrigiano and Nelson, 2004). The neural network operates with powerful compensatory mechanisms, where the inhibition of a targeted node may be rapidly and effectively offset by the heightened activity of redundant or parallel circuits (Krakauer et al., 2017). This network-level compensation means that while a manipulation may successfully alter the target circuit *in vitro* or acutely, the expected behavioral or therapeutic outcome *in vivo* is often muted, transient, or fails entirely, posing a significant barrier to translational efficacy and outcome prediction.

### 2.2 The phenomenological and systems-level gulf

Animal models are essential tools that reduce complex human neuropsychiatric disorders to observable and measurable behaviors, such as “compulsive lever pressing” or “social avoidance.” Although these proxies are operationally useful, they cannot reproduce the inner experience that defines human mental illness—for example, the profound guilt and hopelessness in depression, the unshakable false beliefs in psychosis, or the intrusive and distressing thoughts in obsessive-compulsive disorder (Nestler and Hyman, 2010; Markou et al., 2009). This phenomenological gap is not merely a philosophical distinction but a fundamental scientific constraint: the subjective experience of illness, mediated by higher-order cognitive and introspective circuits in the prefrontal and parietal cortices, is itself a core aspect of pathology that must be understood and addressed. Indeed, many of the constructs we seek to model—such as the negative self-view in depression or the breakdown of reality testing in schizophrenia—are emergent phenomena rooted in human language, abstract reasoning, and socio-cultural context, with no direct counterpart in the animal brain (Miller, , 2010).

This reductionist paradigm critically constrains our circuit-level understanding of brain disorders. The prevailing “one-circuit-at-a-time” framework proves inadequate for capturing the distributed, emergent nature of neural computation. This limitation is vividly exposed by a common dilemma in the literature: for a single disease, multiple studies often each identify a different circuit as “deterministic,” while conversely, the very same circuit is implicated in vastly different—sometimes even opposing—clinical phenotypes. These observations, which appear contradictory under a reductionist lens, are in fact entirely consistent with a network-based understanding of the brain. Rather than operating as a collection of discrete modules, the brain functions as an integrated hierarchical system in which behavior and disease states emerge from dynamic interactions across multiple large-scale networks (Williams, 2016; Mu et al., 2015; Bassett and Sporns, 2017).

Major depressive disorder provides a compelling illustration of this network-level pathology. The clinical presentation extends beyond mere reward circuit dysfunction (e.g., ventral striatal anhedonia) to encompass a hyperactive Default Mode Network (DMN) underlying maladaptive self-referential thought (Hamilton et al., 2011), a dysregulated Salience Network (SN) impairing emotional stimulus detection, and compromised Cognitive Control Networks (CCN) including the frontoparietal network, resulting in executive dysfunction and impaired emotion regulation (Kaiser et al., 2015). Crucially, these networks maintain dynamic equilibrium: failure to suppress the DMN during external task engagement correlates with rumination, while the SN’s inability to facilitate DMN-to-CCN transitions underlies cognitive inflexibility (Menon, 2011). Consequently, targeting isolated nodes—such as augmenting striatal dopamine to alleviate anhedonia—overlooks the dysfunctional network context that generates the pathological state. Such localized interventions frequently prove insufficient, as the core pathology often resides in aberrant interactions between macroscopic networks rather than in any single circuit’s function (Mayberg, 2007).

**FIGURE 1:**
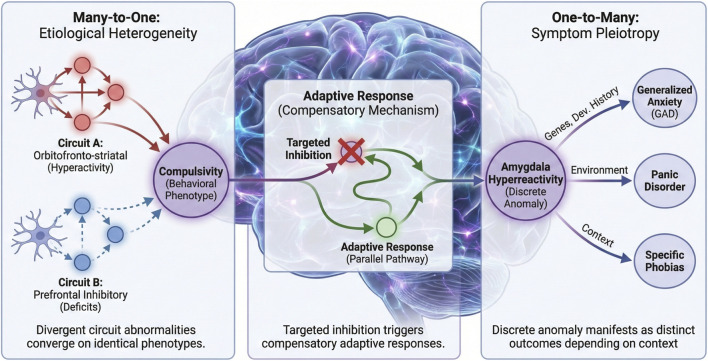
Network-Level Phenomena and the Limitations of Discrete Circuit Modulation. This schematic illustrates the complexity of neural networks as adaptive systems, highlighting why “one-circuit-at-a-time” interventions often fail to produce enduring therapeutic effects. (Left) The “Many-to-One” Principle (Etiological Heterogeneity): Distinct neural circuit abnormalities can converge to produce identical behavioral phenotypes. As shown, disparate mechanisms such as orbitofronto-striatal hyperactivity (top) and prefrontal inhibitory deficits (bottom) can both manifest as the same compulsive behavior observed across OCD and addiction spectra. (Right) The “One-to-Many” Principle (Symptom Pleiotropy): A single discrete circuit anomaly can lead to diverse clinical outcomes depending on the context. Here, amygdala hyperreactivity acts as a core transdiagnostic feature that differentiates into generalized anxiety, panic disorder, or specific phobias, contingent upon modulating factors such as genetic predisposition and environmental history. (Center) Adaptive Response (Homeostatic Plasticity): The central brain network demonstrates the system’s resilience. Targeted inhibition (red cross) of a specific pathological node frequently triggers a compensatory re-routing of neural activity through parallel pathways (green arrow). This adaptive response preserves functional output and contributes to treatment resistance, negating the intended effects of discrete modulation.

Beyond phenomenological and observational limitations, a third fundamental challenge emerges from the brain’s nature as a complex adaptive system. While methodologically convenient, the dominant “one-circuit-at-a-time” investigative approach fundamentally misrepresents the brain’s distributed architecture—a perspective increasingly validated by modern network neuroscience (Bassett and Sporns, 2017). This reductionist vantage point obscures critical network-level phenomena that ultimately determine intervention efficacy. The brain exhibits remarkable homeostatic plasticity and compensatory mechanisms, wherein targeted node inhibition frequently triggers adaptive responses in parallel pathways, preserving functional output and negating intended behavioral effects (Krakauer et al., 2017). This robustness is further complicated by neural systems’ inherent “many-to-one” and “one-to-many” characteristics (Figure 1). The “many-to-one” principle demonstrates how divergent circuit abnormalities—such as orbitofronto-striatal hyperactivity versus prefrontal inhibitory deficits—can converge upon identical behavioral phenotypes like the compulsivity observed across OCD and addiction spectra (Insel and Cuthbert, 2015; Robbins et al., 2012). Conversely, the “one-to-many” principle reveals how a discrete circuit anomaly (e.g., amygdala hyperreactivity) can manifest as clinically distinct outcomes—generalized anxiety, panic disorder, or specific phobias—depending on genetic predisposition, developmental history, and environmental context (Geschwind and Flint, 2015). This explains the apparent paradox: a circuit’s role is defined not by its fixed identity, but by its dynamic state and interactions within the broader network. A hyperactive amygdala may be pathological in one context, yet adaptive in another; its functional impact is contingent upon the network in which it is embedded. Ultimately, both normal cognition and pathological states emerge from this sophisticated systems-level interplay rather than from isolated circuit operations (Bassett and Sporns, 2017; Menon, 2011). This fundamental reality explains why binary “switch”-like circuit manipulations typically achieve only transient benefits, as their effects are inevitably assimilated by the broader network’s dynamic self-organization. This constitutes a fundamental limitation for developing enduring circuit-based therapeutics, pointing to the imperative need for network-resonant interventions over discrete circuit modulation.

### 2.3 The translational validity gulf

#### 2.3.1 Emerging technical solutions for cross-species alignment

The field is evolving strategies to address these challenges through more physiological interventions using temporally precise, pattern-matched stimulation that better mimics natural neural activity (Grosenick et al., 2015; Grossman et al., 2017); cell-type specific targeting that moves beyond regional manipulation to defined neuronal populations and sophisticated experimental designs that incorporate multi-circuit recording during manipulation to understand network-wide effects (Deisseroth, , 2015; Stringer et al., 2019). Furthermore, the recognition that different circuit disturbances may underlie similar behavioral phenotypes in different individuals has led to the important concept of neurobiological subtypes (biotypes) within diagnostic categories. This understanding is driving a shift toward personalized neuromodulation approaches that target specific circuit abnormalities identified through functional neuroimaging rather than applying one-size-fits-all interventions.

Despite these promising advances, fundamental challenges persist. The field still lacks a comprehensive understanding of how circuit manipulations alter overall network dynamics and how these effects evolve over time, severely limiting our ability to predict therapeutic outcomes in humans (Bassett and Sporns, 2017; Calhoun et al., 2014). This knowledge gap between precise circuit-level understanding in animal models and effective translation to human therapies represents the core dilemma of modern translational neuroscience.

#### 2.3.2 Fundamental animal-human biological differences

While the circuit-based understanding of metabolic and neuropsychiatric disorders has been profoundly advanced by rodent models, the translational pathway from bench to bedside is fraught with challenges that stem not from a lack of mechanistic insight, but from the intrinsic limitations of the models themselves and the complexity of the human condition. A primary barrier is the interpretative leap required from conserved circuit motifs in animals to the pathological complexity of the human brain. Although fundamental neural architectures—such as those governing energy homeostasis, fear, or reward—show evolutionary conservation, their instantiation in humans involves a qualitative shift in scale, connectivity, and regulation. For instance, the human brain possesses unique cell types (e.g., von Economo neurons), vastly expanded associative networks, and is modulated by meta-cognitive and sociocultural factors absent in animal models (Hodge et al., 2019; Rilling, 2014). Consequently, a neural node identified as critical for a behavior in a mouse may be embedded within a fundamentally different computational network in humans. This divergence directly implies that a therapeutic intervention effective in a rodent model may, in humans, yield unpredictable efficacy, novel off-target effects, or fail entirely because the targeted node serves a different function within the human connectome (Buckner and Krienen, 2013).

This biological and network-level disconnect is compounded by a fundamental etiological mismatch. Human metabolic and psychiatric disorders are typically polygenic and multifactorial, arising from the interaction of hundreds of genetic risk variants (ranging from hundreds of identified loci in conditions like schizophrenia to an estimated thousands of contributing variants in depression and anxiety) with small effect sizes and cumulative environmental exposures over a lifetime (Geschwind and Flint, 2015; Sullivan et al., 2018). In stark contrast, most definitive preclinical evidence is built upon reductionist models that employ single-gene knockouts, acute environmental stressors, or precise optogenetic manipulations. While these models are indispensable for establishing causality, they create a “causality gap”: the precisely engineered dysfunction that is corrected in the animal may only represent a simplified component of the diffuse, multi-factorial dysregulation that characterizes the human disease (Nestler and Hyman, 2010). Therefore, a therapy that successfully reverses an engineered deficit in a model might not engage the more resilient, heterogeneous pathophysiology in patients.

Furthermore, attempts to bridge this gap using human neuroimaging often reveal inconsistencies that underscore these challenges. For example, neuroimaging biomarkers derived from animal studies—such as hyperactivity in a specific thalamic nucleus—can show variable localization, effect sizes, or even directionality across different human cohorts (Botvinik-Nezer et al., 2020). These inconsistencies often stem from methodological heterogeneities (e.g., differing task paradigms, analytical pipelines), small sample sizes, and the substantial phenotypic and etiological heterogeneity within patient populations diagnosed with the same disorder (Dinga et al., 2019; Hong et al., 2020). Thus, the very tools meant to validate and translate animal findings frequently highlight the complexity they aim to simplify, revealing a landscape where clear, one-to-one mappings between animal circuit pathology and human disease states are the exception rather than the rule (Hyman, 2021).

Perhaps the most significant translational barrier arises from the mismatch between our symptom-based diagnostic systems and the underlying biological reality of mental disorders. Current clinical categories such as DSM-5 or ICD-11 are based on symptom clusters rather than neurobiological mechanisms, meaning that “Major Depressive Disorder” almost certainly represents a heterogeneous umbrella term encompassing multiple distinct pathophysiological conditions that converge on similar symptomatic presentations (Insel and Cuthbert, 2015; Insel et al., 2010). This diagnostic heterogeneity creates a critical problem for translation: a therapy developed to target a specific circuit dysfunction identified in animal models may only be effective for that subset of patients whose depression is actually driven by that particular circuit abnormality. The failure to parse this biological heterogeneity in clinical practice means that clinical trials, which typically enroll patients based on symptom-based criteria rather than circuit-based biomarkers, are likely to fail even when targeting therapies that are genuinely effective for specific neurobiological subtypes (Roet et al., 2020).

This biological and diagnostic divide suggests that successful translation will require not only improved animal models but also a fundamental reconceptualization of psychiatric diagnosis. Transitioning beyond symptom-based categories to circuit-based biomarkers or biotypes may prove essential for identifying patient populations most likely to respond to targeted circuit interventions (Williams, 2016). Furthermore, recognizing the profound biological differences between species should prompt greater caution in extrapolating from animal findings to human applications, while simultaneously motivating the development of a multi-level, complementary translational research framework. On one hand, more human-relevant experimental models should be advanced, such as human stem cell-derived cultures and brain organoids (Pasca, 2018). These systems provide a unique window into human-specific cellular and synaptic pathophysiology, though they currently fall short of recapitulating the complex circuit architecture and behavioral output of an intact organism. On the other hand, validation of circuit mechanisms at the systems level must be strengthened. In this critical step, non-human primates—particularly smaller, genetically modifiable species such as marmosets—constitute an indispensable translational pivot. Their high degree of homology to humans in neuroanatomy, cognition, and social behavior, combined with increasingly sophisticated genetic and circuit-manipulation tools, makes them a powerful platform for the crucial validation of mechanisms and targets discovered in rodents prior to human clinical trials. Therefore, bridging the translational gap demands a coordinated, multi-pronged strategy that leverages the complementary strengths of diverse models while directly confronting the complexity of human disease.

## 3 A path forward: a multi-pronged strategy for translation

The remarkable progress in basic neuroscience over the past two decades has generated unprecedented insights into the neural circuit mechanisms underlying behavior and disease. However, the translation of these fundamental discoveries into effective clinical interventions has proven challenging. This translational gap stems from multiple interconnected limitations in current approaches, including the reductionist nature of circuit manipulation studies, species differences in brain organization, and the heterogeneity of human psychiatric disorders. To bridge this gap, we propose a comprehensive strategy that integrates advances across multiple domains, from novel experimental frameworks and computational approaches to next-generation neuromodulation technologies and interdisciplinary collaboration. Crucially, we acknowledge that this vision, while ambitious, must be pursued with an explicit awareness of practical feasibility, including technological readiness, interdisciplinary integration challenges, and realistic development timelines. This multi-pronged approach aims to transform our understanding of neural circuit function in health and disease and accelerate the development of targeted, effective, and personalized therapies for neuropsychiatric disorders.

### 3.1 From reductionist limits to network-level understanding

The prevailing paradigm in preclinical neuropsychiatry research has been highly successful in establishing causal links between specific neural circuits and behaviors. Using powerful tools like optogenetics, numerous studies have demonstrated that modulating a single pathway can produce a significant behavioral change in animal models. This approach has generated a vast and valuable “map” of potential therapeutic nodes (Deisseroth, , 2015). However, this reductionist framework, while necessary as a first step, presents fundamental limitations that hinder translational progress. By treating neural circuits as independent entities, it fails to account for the complex, dynamic, and non-linear interactions that characterize real brain networks. The approach often overlooks compensatory mechanisms, network-level adaptations, and the emergent properties that arise from interconnected neural systems, leading to potentially misleading conclusions about circuit function and therapeutic potential (Bassett and Sporns, 2017).

A critical limitation is that acute, high-intensity manipulations (e.g., optogenetic “on/off” switching) do not reflect the brain’s natural operating principles, which rely on sparse, asynchronous activity and subtle modulation of firing patterns (Grosenick et al., 2015). Such non-physiological interventions can force artificial behavioral states that bypass the very network dynamics we aim to correct. Furthermore, the brain exhibits remarkable resilience through homeostatic plasticity and functional redundancy. The inhibition of a critical node may be rapidly compensated for by increased activity in parallel or alternative pathways, a phenomenon observed in both motor and limbic systems, which can mask the intended effect of a manipulation and lead to transient therapeutic outcomes (Bargmann and Marder, 2013).

Therefore, a fundamental shift is urgently needed—from a circuit-centric to a network-centric perspective. This requires the adoption of “manipulate-and-record” experimental designs, where a specific circuit is perturbed while simultaneously monitoring the resulting changes in activity across multiple brain-wide networks using techniques such as large-scale electrophysiology or calcium imaging (Hausser, 2014). This approach allows researchers to move beyond asking “What behavior does this circuit control?” to the more systems-level question: “How does perturbing this node alter the entire network’s functional configuration, and how do these network-level changes correlate with behavior?”

### 3.2 Integrating dynamic network principles and personalized therapies

Building on this network-centric view, the next frontier is not to simply catalogue more circuits but to understand their synergistic and antagonistic interactions within the broader network context. The key question is no longer “Does this circuit regulate behavior?” but rather “How does modulating this circuit alter the broader network dynamics to produce a behavioral change, under what conditions is this modulation effective, and how can we strategically target multiple nodes to achieve synergistic, long-lasting effects while minimizing compensatory adaptations?”

This requires moving from open-loop, single-target manipulation towards closed-loop, systems-level approaches that can read out and manipulate the network state in real-time (Grosenick et al., 2015; Ferrero et al., 2025). For instance, rather than continuously stimulating a region, a closed-loop system would detect a specific, pathological network oscillation (e.g., in the amygdala-prefrontal cortex circuit) and deliver a precisely timed pulse to disrupt it, thereby guiding the network back to a healthy state. Such approaches would integrate neurotechnology, computational modeling, and control theory to understand how interventions perturb overall brain dynamics and how these dynamics evolve over time (Bassett and Sporns, 2017).

However, the implementation of such closed-loop neuromodulation and network control strategies faces significant practical challenges. First, it requires a substantial interdisciplinary infrastructure, integrating advanced neural interface hardware capable of high-fidelity recording and stimulation, real-time biomarker detection algorithms, and control-theoretic models robust to individual neuroanatomical and dynamic variability. Second, the development and regulatory approval of adaptive, algorithm-driven devices present a more complex pathway than traditional open-loop systems, necessitating new clinical trial designs and safety standards (Frank et al., 2019; Musk and Neuralink, 2019; Widge and Sahay, 2016). Realistically, widespread clinical deployment is a long-term goal, likely proceeding through iterative phases: (1) validation in highly controlled, invasive settings for refractory conditions (e.g., next-generation adaptive DBS for Parkinson’s disease) (Little et al., 2013), followed by (2) gradual translation to less invasive platforms (e.g., EEG-triggered TMS) for broader neuropsychiatric applications over the next decade.

To realize this vision, a corresponding evolution in basic neuroscience is essential. This paradigm shift necessitates the development of new experimental frameworks capable of simultaneously monitoring and manipulating multiple circuit elements while tracking distributed network-wide responses. Complementing this, advanced computational methods—such as network control theory—are needed to identify critical control points and predict the system-wide effects of interventions (Gu et al., 2015). The overarching goal is to progress from mapping static anatomical connections to deciphering the brain’s dynamic “traffic laws”—the principles governing real-time information flow and interactions within neural populations (Taylor et al., 2018; Bazinet et al., 2023).

Ultimately, this integrated understanding will enable a new therapeutic logic. We will move beyond identifying “which buttons to push” to knowing “when, how, and in what sequence to push them” to achieve desired outcomes while preserving system stability. Crucially, this framework enables precision neuromodulation. By using functional neuroimaging to define an individual’s unique “network fingerprint” of dysfunction (Mohammad et al., 2025), therapies can be tailored to target specific pathological dynamics. For example, depression in one patient may be driven primarily by hyperconnectivity within the Default Mode Network, while in another it may stem from hypoactivity of the Cognitive Control Network. Consequently, stimulating the same anatomical target (e.g., the subgenual cingulate) may require fundamentally different parameters to normalize these distinct network abnormalities (Bormann et al., 2021; Klooster et al., 2022; Fox et al., 2012). This evolution from a one-size-fits-all, anatomy-centric approach to a dynamic, circuit-informed, and personalized strategy promises to deliver therapies that are more effective, durable, and precisely aligned with an individual’s pathophysiology.

### 3.3 Back-translation and dimensional approaches

This conceptual evolution naturally leads to practical implementation strategies that must be guided by a deliberate translational mindset. Recent proposals emphasize the need for a paradigm shift. For instance, the “shiftability” paradigm advocates designing preclinical studies to explicitly test the malleability of a neural or behavioral trait—akin to testing a clinical intervention’s potential—rather than merely cataloging static deficits (Whelan et al., 2024). This approach directly addresses the ecological validity gap. Concurrently, aligning the concepts of replication across basic and clinical neuroscience is critical (Lewis and Nobis, 2023). While basic science seeks to replicate precise mechanistic causality under controlled conditions, clinical translation requires demonstrating robust, generalizable effects in heterogeneous populations. Acknowledging and designing for this distinction—pursuing both “mechanistic replicability” and “clinical generalizability”—is essential for building a credible translational pipeline.

A fundamental shift toward “back-translation” represents a critical starting point, moving beyond traditional forward translation by initiating research with human circuit-based biomarkers (Williams, 2016; Xia and Kheirbek, 2020). Through advanced neuroimaging techniques including high-field functional MRI, diffusion tensor imaging, magnetoencephalography, and intracranial recordings, researchers can identify specific neural signatures of pathology—such as distinctive functional connectivity patterns between prefrontal and limbic regions in treatment-resistant depression (Rengasamy et al., 2024; Lui et al., 2011; Morriss et al., 2024). These human-derived biomarkers subsequently serve as blueprints for creating mechanistically grounded animal models, where optogenetic and chemogenetic tools enable precise recreation of human circuit dynamics (Calhoon and Tye, 2015). This approach establishes robust causal relationships between network abnormalities and behavioral outcomes, ensuring that basic research remains firmly anchored in human neurobiology.

Building upon this human-data-driven foundation, the field must embrace dimensional behavioral constructs that transcend conventional disease categories and species boundaries. The Research Domain Criteria framework provides a valuable structure for this transition, focusing investigation on conserved neurobehavioral dimensions including threat reactivity, reward valuation, and cognitive control (Cuthbert and Insel, 2013; Morris et al., 2022). By developing analogous behavioral paradigms across species—such as probabilistic reversal learning tasks for cognitive flexibility or social defeat paradigms for social avoidance—researchers can establish direct experimental bridges between animal circuit manipulation and human brain function (Osakada et al., 2024; Hu et al., 2021; Li M. et al., 2025). This dimensional approach not only facilitates more precise translation but also acknowledges the substantial heterogeneity within traditional diagnostic categories, allowing for more personalized therapeutic strategies that target an individual’s specific core dysfunction. The identification of these core dysfunctions is increasingly powered by data-driven approaches. Mining large-scale human genomic and transcriptomic datasets (e.g., from post-mortem brain tissue or peripheral biomarkers) can reveal convergently dysregulated molecular networks that cut across diagnostic categories, providing high-value, clinically-anchored targets for mechanistic study (O'Connor et al., 2023). To bridge the gap between these molecular/circuit targets and whole-organism clinical outcomes, Quantitative Systems Pharmacology modeling offers a powerful computational framework (Bloomingdale et al., 2021). By integrating multi-scale data—from molecular pathways and neural circuit dynamics to behavior and clinical symptoms—QSP can construct “*in silico* patient” models. These models can simulate disease progression, predict differential intervention outcomes for patient subtypes, and optimize clinical trial design, thereby de-risking the translational pathway for circuit-based discoveries and paving the way for personally tailored therapeutic strategies that target an individual’s specific core dysfunction, rather than a broad diagnostic label (Williams, 2016; Tozzi et al., 2024).

### 3.4 Advanced neuromodulation and integrated team science

The insights gained from human-relevant circuit manipulations and cross-species behavioral assessments are critically informing the development of a new generation of neuromodulation technologies (Krauss et al., 2021; Pereira et al., 2024; Lisoni and Barlati, 2025; Horn and Neumann, 2025) (Table 3). While current clinical tools like transcranial magnetic stimulation (TMS) and deep brain stimulation (DBS) have demonstrated therapeutic value, they face significant limitations in spatial precision, depth penetration, and mechanistic specificity. TMS is largely restricted to superficial cortical regions, and DBS, though effective for some conditions, requires invasive neurosurgery with associated risks (van Rooij and Holtzheimer, 2019; Pitcher et al., 2021). Emerging technologies are now offering promising pathways to overcome these challenges by enabling non-invasive access to deep brain circuits and delivering more adaptive, physiologically-informed interventions.

**TABLE 3:** A summary of new generation of neuromodulation technologies.

Technology	Principle of action	Key advantages/Innovation	Ref.
Focused Ultrasound (tFUS)	Uses acoustic energy focused through the skull to modulate activity in deep brain structures	Non-invasive access to deep brain regions (e.g., limbic structures) without neurosurgery; high spatial precision	Bystritsky et al. (2011), Tufail et al. (2010)
Temporal Interference (TI) Stimulation	Applies multiple high-frequency electric fields that only interfere constructively within a targeted deep region	Non-invasive deep brain modulation that bypasses superficial cortical areas, increasing focal specificity	Grossman et al. (2017), Esmaeilpour et al. (2021), Liu et al. (2024)
Transcutaneous Auricular Vagus Nerve Stimulation (taVNS)	Stimulates the auricular branch of the vagus nerve (in the ear) using surface electrodes	Accessible, non-invasive alternative, modulates central nervous system activity and neuro-immune interactons	Yap et al. (2020), Butt et al. (2020), Frangos et al. (2015), Kaniusas et al. (2019)
Closed-Loop Systems (BCIs)	Continuously monitor neural biomarkers (e.g., LFP oscillations, firing patterns) and deliver adaptive, precisely timed intervention only when a pathological state is detected	Paradigm shift from continuous open-loop: aims to restore naturalistic brain dynamics. Enhanced efficacy and tolerability	Berenyi et al. (2012), Krook-Magnuson et al. (2015)
Closed-Loop Brain-Body Interfaces	Integrates central neural signals with peripheral physiological data or nerve stimulation	Holistic and adaptive neuromodulation by accounting for systemic physiological state alongside brain activity	Yu et al. (2025), Sun et al. (2022a), Shirvalkar et al. (2018), Sun et al. (2022b)

A key frontier in translating circuit-based insights is the development of non-invasive deep brain stimulation techniques that can modulate subcortical circuits with spatial precision. Low-intensity transcranial focused ultrasound (tFUS) represents a particularly promising modality, as it uses acoustic energy to directly and focally modulate deep brain structures without surgical intervention (Grossman et al., 2017; Legon et al., 2014; Barksdale et al., 2025). Early proof-of-concept studies have demonstrated its feasibility for modulating mood and behavior in humans by targeting limbic structures (Sanguinetti et al., 2020). However, translating this promise into routine therapy requires overcoming significant hurdles. The long-term biological effects of repeated ultrasound energy on neural tissue, glia, and the blood-brain barrier remain areas of active investigation, as do the optimization of treatment parameters (e.g., dosing, frequency) for specific disorders. Furthermore, the path to widespread clinical deployment will necessitate addressing challenges related to device standardization, operator training, and the completion of large-scale, randomized, sham-controlled trials to definitively establish efficacy and safety—a regulatory and financial undertaking typical of Class II/III medical devices.

Similarly, temporal interference (TI) stimulation offers a novel electromagnetic approach by using multiple, high-frequency electric fields that interfere constructively only within a targeted deep brain region (Xu et al., 2025; Hummel, 2025), thereby achieving focal neuromodulation without directly stimulating overlying cortical areas (Grossman et al., 2017). While the physics is elegant, its therapeutic scalability faces practical questions about achieving sufficient focality and depth in the heterogeneous human brain, as well as the need for personalized dosing models. Transcutaneous auricular vagus nerve stimulation (taVNS) has emerged as a more accessible means to modulate central nervous system activity and neuro-immune interactions via the auricular branch of the vagus nerve, showing preliminary potential for treating depression and inflammatory conditions (Zou et al., 2024; Wang et al., 2021). Its primary challenge lies not in spatial precision but in protocol optimization and mechanistic specificity; determining reliable dosing parameters and understanding how peripheral stimulation translates to central therapeutic effects are critical for validating it as a robust circuit-targeted intervention rather than a general neuromodulator.

The most transformative advance, however, may be the rise of closed-loop neuromodulation systems, which represent a true paradigm shift from traditional open-loop approaches (Afshar et al., 2012). These systems, often conceptualized as brain-computer interfaces (BCIs) (Edelman et al., 2025; Zhang et al., 2024; Liu et al., 2025), continuously monitor neural activity biomarkers—such as specific local field potential oscillations or aberrant firing patterns—and deliver precisely timed, adaptive interventions only when a pathological state is detected (Widge et al., 2018; Scangos et al., 2021). This approach aims to restore naturalistic brain dynamics, potentially enhancing both efficacy and tolerability by avoiding continuous stimulation (Widge et al., 2019). The concept is now expanding to include closed-loop brain-body interfaces, which integrate central neural signals with peripheral physiological data or nerve stimulation to achieve more holistic and adaptive neuromodulation (Yu et al., 2025). These advanced neuromodulation tools hold immense promise for psychiatry. By directly targeting the circuit-specific biomarkers identified through the back-translational and dimensional research strategies outlined earlier, they offer a path toward truly mechanism-based, personal ized treatments. This convergence of basic neuroscience and advanced engineering has the potential to revolutionize therapy for refractory neuropsychiatric conditions such as depression, OCD, and PTSD.

Ultimately, realizing the full potential of these technological and methodological advances demands the dissolution of traditional academic silos through genuinely integrated, team-based science. The profound complexity of neural circuit dysfunction in psychiatric disorders—spanning molecular, cellular, systems, and behavioral levels—necessitates collaborative teams that unite clinical neuroscientists, engineers, computational biologists, and data scientists from the initial project conception through to final implementation and validation (Insel and Landis, 2013; Siebner et al., 2009; Verdonk et al., 2025; Wool and International Brain, 2020). This deep integration creates a seamless, iterative pipeline from bedside to bench and back again, ensuring that therapeutic development is both biologically grounded and clinically relevant. This collaborative cycle begins with clinicians and human neuroscientists identifying circuit-based biomarkers in patient populations using advanced neuroimaging and electrophysiology (Xia and Kheirbek, 2020). These empirical findings are then translated into causal, testable hypotheses by computational neuroscientists, who develop dynamic network models to formalize the dysfunction and identify critical control points for intervention (Bassett and Bullmore, 2017). Engineers subsequently leverage these mechanistic insights to create and refine novel neuromodulation technologies, such as optimizing the dosing and targeting parameters for non-invasive approaches like taVNS to enhance their specificity and efficacy (Matsuoka et al., 2025; Sigrist et al., 2023). Basic scientists then rigorously validate these targeted interventions and their underlying mechanisms in genetically tractable animal models, which in turn refines the human biomarkers and computational models, creating a virtuous cycle of discovery (Mathis et al., 2024; Miehl et al., 2023). This iterative team science approach, fundamentally rooted in the principles of translational psychiatry, ensures that therapeutic development remains firmly grounded in clinical reality while simultaneously leveraging the full power of modern neuroscience, engineering, and data science to create effective, biomarker-driven treatments for neuropsychiatric disorders.

### 3.5 Re-engineering the research ecosystem: aligning culture with translation

The ambitious technical and collaborative strategies outlined here will inevitably falter without a parallel revolution in academic culture. Modern systems neuroscience faces a profound paradox: we have mastered the art of producing dazzling, publication-ready circuit discoveries while remaining remarkably inept at translating them into clinical solutions. This is not a failure of scientific imagination, but of systemic incentive design—an academic economy that systematically prioritizes technical novelty over therapeutic impact, visual elegance over clinical relevance, and mechanistic cleverness over translational durability.

This preference permeates our literature and shapes its priorities. High-impact journals are filled with technically exquisite demonstrations—multimodal imaging paired with elegant circuit manipulations in homogeneous models—crafted to maximize visual impact and conceptual novelty. While scientifically rigorous, these “high-concept” studies often prioritize aesthetic appeal and theoretical innovation over practical translatability. Concurrently, the essential but unglamorous work that forms the true bedrock of translation—stress-testing findings across disease models and developmental stages, optimizing real-world intervention parameters, and publishing rigorous negative replications—remains chronically undervalued and underfunded. We have perfected the production of technically sophisticated, visually compelling mechanistic narratives optimized for high-profile publication, while neglecting to build the translational infrastructure needed to convert these narratives into reliable therapies.

This systemic preference creates what we term the “reproducibility-translation chasm.” An elegant optogenetic demonstration that produces striking behavioral effects under tightly controlled conditions—often celebrated in prestigious publications—may fail in slightly different experimental setups or, more critically, may not engage comparable network dynamics in the heterogeneous human brain with its lifetime of unique experiences. Yet our academic reward system continues to incentivize the discovery of visually impressive, conceptually novel circuits over the rigorous validation of existing ones for therapeutic potential. The result is a literature rich in technical virtuosity but shallow in translational depth, where the path from a Nature cover story to a Phase I clinical trial remains largely uncharted.

To bridge this chasm, a fundamental recalibration of our academic value system is urgently needed. This requires concerted, structural reforms across key stakeholders. Funding agencies could play a pivotal role by establishing dedicated programs that explicitly prioritize and reward clinical translation—valuing the robust validation of biomarkers and target engagement as highly as novel mechanistic insight. Similarly, journals, especially those at the neuroscience-clinical interface, have a unique opportunity to lead by championing new article formats. Introducing categories such as “Translation-Ready Reports”—which would require authors to outline feasible, staged paths to clinical intervention—could incentivize and elevate research with clear translational trajectories. Perhaps most critically, academic institutions need to reform promotion and tenure criteria to properly recognize the multidisciplinary architects of translation. The contributions of engineers, data scientists, and clinician-scientists who bridge foundational discovery and therapeutic application should be valued, with patents, successful team leadership, and regulatory milestones considered alongside traditional publication metrics. In this reconfigured framework, the impact of a circuit discovery would be judged not solely by its technical elegance or conceptual novelty, but equally by the clarity and credibility of its translational pathway.

## 4 Conclusion

The translational dilemma in neural circuit research, while formidable, is surmountable through the deliberate adoption of concrete, integrated strategies that move beyond generic calls for “better approaches”. To bridge the translational gap, the field must adopt an integrated strategy beginning with the establishment of international, open-science Circuit Biomarker Consortia to define standardized, human-derived circuit-behavioral biomarkers (e.g., amygdala-prefrontal connectivity in threat processing) as mandatory benchmarks for preclinical validation. Building upon these biomarkers, preclinical research must systematically implement a “Back-Translation” Pipeline, wherein etiologically complex animal models (e.g., polygenic or chronic stress paradigms) are explicitly designed to recapitulate specific circuit dysfunctions identified in patients, thereby creating a closed, iterative loop between human observation and mechanistic experimentation. Concurrently, achieving true cross-species alignment necessitates a fundamental shift from modeling syndromic diagnoses to targeting evolutionarily conserved behavioral and physiological dimensions (e.g., anhedonia, cognitive control), incentivized through grant reviews and the development of trans-diagnostic, dimensionally-stratified cohorts. Finally, the clinical pathway must accelerate toward personalized, closed-loop neuromodulation, driven by increased investment in adaptive devices and trials of algorithm-driven stimulation that adjusts in real-time based on individual neural biomarkers. The synergistic implementation of these interconnected pillars—biomarker standardization, etiologically-informed modeling, dimensional phenotyping, and adaptive intervention—can transform the translational pipeline into a virtuous cycle, converting today’s scientific promise into tomorrow’s clinically meaningful outcomes for brain disorders.

The promise of circuit-based therapeutics will be realized not through isolated advances, but by the synergistic implementation of this framework. By anchoring research in human-derived benchmarks, enforcing etiological validity in models, focusing on conserved dimensions, and engineering adaptive interventions, we can transform the translational pipeline into a virtuous cycle. Ultimately, this endeavor requires more than methodological shifts; it demands a cultural commitment to genuinely collaborative team science that breaks down traditional barriers between clinics, laboratories, and engineering hubs. By recognizing that the translational impasse stems from the profound challenge of bridging levels of biological organization, and by uniting behind these actionable strategies, we can aim to transform the present period of challenges into a historic inflection point—one that finally delivers on the long-deferred promise of targeted, effective, and personalized interventions for brain disorders.
